# A Review of Circumpolar Arctic Marine Mammal Health—A Call to Action in a Time of Rapid Environmental Change

**DOI:** 10.3390/pathogens12070937

**Published:** 2023-07-14

**Authors:** Ashley Barratclough, Steven H. Ferguson, Christian Lydersen, Peter O. Thomas, Kit M. Kovacs

**Affiliations:** 1 National Marine Mammal Foundation, 2240 Shelter Island Drive, San Diego, CA 92106, USA; 2 Arctic Aquatic Research Division, Fisheries and Oceans Canada, Winnipeg, MB R3T 2N6, Canada; steve.ferguson@dfo-mpo.gc.ca; 3 Norwegian Polar Institute, Fram Centre, 9296 Tromsø, Norway; christian.lydersen@npolar.no (C.L.); kit.kovacs@npolar.no (K.M.K.); 4 Marine Mammal Commission, 4340 East-West Highway, Room 700, Bethesda, MD 20814, USA; pthomas@mmc.gov

**Keywords:** *Brucella*, cetaceans, emerging infectious diseases, influenza A virus, morbillivirus, pinnipeds, polar bear, seals, *Toxoplasma gondii*, whales

## Abstract

The impacts of climate change on the health of marine mammals are increasingly being recognised. Given the rapid rate of environmental change in the Arctic, the potential ramifications on the health of marine mammals in this region are a particular concern. There are eleven endemic Arctic marine mammal species (AMMs) comprising three cetaceans, seven pinnipeds, and the polar bear (*Ursus maritimus*). All of these species are dependent on sea ice for survival, particularly those requiring ice for breeding. As air and water temperatures increase, additional species previously non-resident in Arctic waters are extending their ranges northward, leading to greater species overlaps and a concomitant increased risk of disease transmission. In this study, we review the literature documenting disease presence in Arctic marine mammals to understand the current causes of morbidity and mortality in these species and forecast future disease issues. Our review highlights potential pathogen occurrence in a changing Arctic environment, discussing surveillance methods for 35 specific pathogens, identifying risk factors associated with these diseases, as well as making recommendations for future monitoring for emerging pathogens. Several of the pathogens discussed have the potential to cause unusual mortality events in AMMs. *Brucella*, morbillivirus, influenza A virus, and *Toxoplasma gondii* are all of concern, particularly with the relative naivety of the immune systems of endemic Arctic species. There is a clear need for increased surveillance to understand baseline disease levels and address the gravity of the predicted impacts of climate change on marine mammal species.

## 1. Introduction

Environmental change resulting from climate warming is well documented, particularly in the Arctic, where increases in air and water temperatures and concomitant reductions in sea ice have been dramatic over recent decades [1]. These physical changes to the environment have the potential to impact the health of Arctic marine mammals (AMM) [2,3,4,5]. Reductions in the extent, seasonal duration of coverage, and thickness of sea ice can have direct consequences on marine mammals by impacting mating, offspring rearing, and foraging, as well as habitat availability and ultimately species distributions and abundances [6,7,8,9,10]. Similar to other animals in the Arctic, AMMs are highly specialised to deal with both high levels of interannual variation and what are for many other species harsh environmental conditions [11]. These specialised adaptations to unique Arctic environments have resulted in relatively low species diversity, which in turn leads to modestly simple food webs and also minimal interspecific competition [2]. As K-selected species, AAM reproductive rates are slow, limiting their rates of population growth or recovery [12,13]. Additionally, they have had limited exposure to diseases because of the harsh environment and little contact with broad-ranging species. Ecosystem disruptions such as the major habitat changes currently taking place in the Arctic can precipitate an increase in disease in wildlife through shifts in individual immunity and pathogen evolution and transmission [14,15,16,17,18]. Individual susceptibility to disease and the ability of species to adapt to the changing environmental conditions will ultimately influence their long-term survival [19,20,21].

Potential emerging health concerns in AMMs that could be driven by climate change are plentiful. One of the biggest risks to AMMs is the naiveté of these populations to pathogen exposure and their subsequent limited immunity. Emerging infectious diseases in naïve populations can result in rapid population declines [22]. The migration of temperate species into the Arctic could result in the arrival of novel pathogens, for example phocine distemper virus or cetacean morbillivirus, which could result in widespread morbidity and mortality. AMMs should be monitored for emerging health concerns, but the challenges are significant because of their remoteness, the extreme environmental conditions, and their dispersal over vast areas. Major mortality events could go undetected in many AMM populations. Baseline serologic data could provide documentation of the presence or absence of disease as well as allowing for the monitoring of increases or decreases in disease exposure or prevalence. Biological health assessment data such as serum biochemistry have been published for several AMMS, providing baseline data for “normal” values for these species [23,24,25,26,27,28,29]. Other biomarkers such as haptoglobin and cortisol have also been measured in ringed seals (*Pusa hispida*), narwhals (*Monodon monoceros*), and white whales (*Delphinapterus leucas*) [30,31,32]. Monitoring of current health and mortality trends in the 11 endemic Arctic species is essential to determine health status and to detect change [33]. An interdisciplinary approach combining longitudinal health data from citizen science observations (including indigenous hunters), governmental research programs, and international scientific collaborations could be employed to achieve a holistic approach to wildlife health monitoring and successful species conservation [34]. 

Health cannot be defined purely from an absence of disease. Holistic assessments need to encompass behaviour, movement, and reproduction, as well as physical and psychological wellbeing [35]. Such assessments are challenging to perform on free-ranging marine mammals; post-mortem examinations are often the only available proxy to assess marine mammal health in many species [16,36,37]. Mortalities of marine mammals above expected rates or in abnormal circumstances are referred to as unusual mortality events (UMEs) [38]. While mass mortalities in terrestrial mammals can be observed relatively easily in most species, marine mammal carcasses often go undetected because of sinking. Additionally, in the case of AMMs, detections are challenging even if carcasses wash ashore, since human populations in Arctic areas are small and not widely distributed, and polar bears (*Ursus maritimus*) and other scavengers patrol coastal areas for carrion [39]. A lack of basic data on population size, reproduction, and mortality makes confirming UMEs challenging. Despite these challenges, several recent UMEs have been detected in the Pacific Arctic. A Pacific walrus (*Odobenus rosmarus*) UME was declared in 2011, predominantly consisting of calves with unusual ulcerated skin lesions leading to the case definition of “Pacific walrus ulcerative dermatitis syndrome”; however, environmental factors and underlying pathogens were not determined [40]. An ice-seal UME from 2011–2016 included ringed, bearded (*Erignathus barbatus*), ribbon (*Histriophoca fasciata*), and spotted seals (*Phoca largha*) predominantly in northwestern Alaska with at least 657 recorded deaths (https://www.fisheries.noaa.gov/alaska/marine-life-distress/diseased-ice-seals-and-unusual-mortality-events, accessed on 12 December 2022). The seals had skin lesions, and the histopathological findings included hepatic abnormalities including lymphoplasmacytic hepatitis and lobular necrosis, bronchopneumonia, *Otostrongylus circumlitus,* and mild myocardia necrosis [41]. Clinically, in the ringed seal cases, where blood sampling was possible, bilirubin and alanine transaminase (ALT) were elevated in comparison to published baseline values, confirming hepatic pathology. However, despite extensive investigations, including ruling out pathogens such as poxvirus, herpesvirus, papillomavirus, morbillivirus, and calicivirus, the underlying cause of the UME was not determined [41]. Cases were documented from Alaska to Canada and Russia, indicating this was not an isolated incident within a small subpopulation [42]. Starting in June 2018, a second UME has been declared in Alaska involving stranded ice seals, with 368 seals found dead as of 07 January 2022 [43]. Similar to the first UME, the underlying cause has not yet been determined (https://www.fisheries.noaa.gov/ accessed on 5 November 2022).

A recent UME involving cetaceans has also been documented, on a much smaller scale, involving bowhead whales (*Balaena mysticetus*) in the eastern Canada–west Greenland stock, with eleven whales found dead in a seven month period beginning October 2020 [44]. The causes of death are unknown, in part because of limited access to be able to perform necropsies. Although the number of carcasses found to date is relatively small, it is extremely unusual to find a cluster of dead bowhead whales. Another cetacean-related UME has also been declared for grey whales (*Eschrichtius robustus*). While grey whales are not a species endemic to the Arctic, their summer migration does extend into Conservation of Arctic Flora and Fauna (CAFF) Arctic boundaries. Since January 2019, 133 dead grey whales have been found in Alaska. The cause of the mortality is unknown, but many whales were in a poor body condition with a few considered to be emaciated (https://www.fisheries.noaa.gov/national/marine-life-distress/2019-2023-gray-whale-unusual-mortality-event-along-west-coast-and#gray-whale-strandings (accessed on 14 march 2023)) [45]. 

The lack of baseline monitoring data on health and disease in AMMs limits our ability to predict or assess the outcome of a changing climate on their health status. However, we can extrapolate from the available literature on other species and from model predictions to make recommendations for sampling approaches in the future to fill data gaps [46]. The focus of this review is primarily on the 11 AMMs, as identified by Conservation of Arctic Flora and Fauna (CAFF), namely three cetaceans (narwhal, white whales, bowhead whale), seven pinnipeds (bearded, harp (*Pagophilus groenlandicus*), hooded (*Cystophora cristata*), ribbon, ringed, and spotted seals, and walruses, as well as the polar bear. In addition, the harbour seal (*Phoca vitulina;* [47]), grey seal (*Halichoerus grypus*; [48]) and harbour porpoise (*Phocoena phocoena*), which generally have more temperate distributions, are included because they have populations that reside within the CAFF boundaries. Additionally, some attention is devoted to migratory whales that are increasingly occurring in some Arctic areas [49,50,51,52,53] because of the risk they pose regarding disease transmission to AMMs [54]. The species include blue (*Balaenoptera musculus*), fin (*Balaenoptera physalus*), grey, humpback (*Megaptera novaeangliae*), killer (*Orcinus* orca), sperm (*Physeter macrocephalus*), minke (*Balaenoptera acturostrata*), and northern bottlenose (*Hyperoodon ampullatus*) whales and white-beaked dolphins (*Lagenorhynchus albirostris*) [52,55,56,57,58,59]. 

This review highlights: (1) the presence of disease documented in AMMs; (2) the risk factors associated with these diseases and emerging infectious diseases; (3) trends in disease transmission (where possible); and (4) recommendations for future monitoring, taking into account the hotspots of marine mammal aggregation identified in Hamilton et al. (2022) [60].

## 2. Materials and Methods

Searches were conducted in the peer-reviewed published literature and in governmental reports for the 23 marine mammal species identified above for the following topics: climate change, AMM health and pathology. In addition, key words for 35 possible pathogens that might impact AMMs were searched for independently and in conjunction with each species. Finally, the literature cited in the articles and book chapters found through the above searches was examined. Where appropriate, the PRISMA guidelines (Preferred Reporting Items for Systematic Reviews and Meta-Analyses) were followed for protocol, search strategy, and risk of bias assessment [61]. The influences of toxins or contaminants on AMM health were not included in this study. The St Lawrence Estuary white whale stock (which is found south of the CAFF Arctic boundaries) did not receive focussed attention because contaminants played such a major role in earlier health issues for this southern population [62,63]. Non-infectious causes of disease and direct anthropogenic effects on health such as Arctic industrial development, increased shipping traffic resulting in trauma, or increased fishing practices increasing entanglements are only briefly summarised herein as contributors to cumulative impacts, because other recent publications address these causes of mortality in relation to climate change [64,65,66,67,68].

## 3. Results

Pathogens were reviewed according to their classification as bacterial, fungal, parasitic, protozoal, and viral agents. The 35 pathogens that were assessed to be of particularly high relevance to the selected marine mammal species are listed in Supplementary Table S1. All these pathogens can infect all AMMs (unless specifically stated otherwise). 

### 3.1. Bacteria

Improved and new diagnostic techniques such as PCR (polymerase chain reaction) are improving bacterial detection methods, and genomic sequencing is now enabling bacterial characterisation. The primary challenge in the assessment of the potential impacts of bacteria on AMMs is determining whether their presence is pathogenic or if they are normal commensals. Below, the most clinically relevant bacterial diseases for potential impacts for AMMs are reported. 

#### 3.1.1. *Brucella*

*Brucella* is a Gram-negative coccobacillus, which is known to cause abortion and infertility in livestock. *Brucella* has been reported in marine mammals, where it can cause sporadic late-term foetal-loss in cetaceans [69,70,71,72,73]. *Brucella ceti* and *B. pinnipedialis* are emerging concerns in marine mammal populations around the world, with the two different strains being observed in cetaceans and pinnipeds, respectively. *B. pinnipedialis* appears to be endemic within many seal populations with minimal pathological effects observed [74,75]. *Brucella* is of particular concern in already at-risk species due to its potential to cause reproductive failure [76]. *Brucella* can be transmitted through contact with an infected individual via respiratory exposure through damaged mucosa or via skin as well as through the ingestion of infected material or transmission through copulation or lactation [77]. Increased densities of animals, for example through a reduction in haul-out sites or the size of breeding areas, resulting in closer contact between individuals, could result in increased transmission of *Brucella.* Recent suggestions of lungworms carrying *Brucella* spp. could provide another route of transmission to AMMs through the consumption of intermediate host fish species [78,79,80]. Clinical signs in addition to abortion are primarily neurological symptoms with meningitis, meningoencephalitis, choroiditis, and abnormal cerebrospinal fluid (CSF) noted upon post-mortem examination [81,82]. 

The detection of *Brucella* is undertaken via isolation or PCR from tissue samples post-mortem or via blood sampling from live individuals and serum screening via competitive ELISA [83,84,85]. Isolation and characterization remain the gold standard. Extensive research has been conducted to identify the best methods to detect *Brucella* isolates, with an emphasis on sequence-base approaches, namely MLVA (multiple locus variable-number tandem repeats analysis), MLSA (multi-locus sequence analysis), SNP (single nucleotide polymorphisms)-typing, and whole-genome analysis [76]. 

In the Arctic, polar bears have been confirmed to be positive for *Brucella* spp. with a prevalence of 5.4% of anti-*Brucella* antibodies in a recent study screening 297 bears from Svalbard and the Barents Sea and in a study in the southern Beaufort Sea, showing a seroprevalence of 10% [86,87,88]. Evidence of reproductive disorders attributed to *Brucella* in these populations has not been documented [86]. Polar bear blood samples from the southern Beaufort Sea (N = 139) were tested for exposure to *Brucella,* which increased with time through the data series and was 2.5 times less likely for more terrestrial bears in their sample compared to bears that spent more time on the sea ice [89]. Sera from several Arctic seals have also been found to be positive for *Brucella*, with the following prevalences: hooded seals (35%); harp seals (2%); and ringed seals (10%) [90,91,92]. Given that seals are an important food source for polar bears, this is the most likely route of transmission for polar bears [90]. In a longitudinal study sampling harbour seals in southeast Alaska from 1976–1999, 46% were found to have antibodies to *Brucella* with antibody prevalence increasing with age [93]. Similarly, *Brucella* was isolated from bearded seals in Svalbard and Alaska [83,94] and in Baltic ringed seals [95], and anti-*Brucella* antibodies have been detected in Atlantic walruses [75,96]. In cetaceans, antibodies for *Brucella* have been found in minke whales (8 %) and fin whales (11%) [90]. White whales in Svalbard and in the Russian Arctic have also been found to be positive for *Brucella* antibodies [97]. A serological screening of white whales from Bristol Bay and the Chukchi Sea confirmed the presence of *Brucella* antibodies, indicating widespread exposure to the bacteria. However, the low level of rtPCR and lack of positive culture results demonstrated that clinical Brucellosis was not prevalent in Alaska [98,99]. Blood samples from 339 narwhal samples between 1984 and 2003 confirmed antibody presence to *Brucella* [100]. However, currently there are no reports of mortality attributed to this bacterium.

*Brucella* is more frequently reported from temperate climates; however, this could be due to higher levels of monitoring rather than higher prevalence [82,101,102,103]. Nevertheless, obtaining baseline *Brucella* levels in AMMs is important to determine if levels increase in the future, particularly if climate change is favourable to *Brucella* [74]. *Brucella* is a primary emerging pathogen of concern for bowhead whales as it has not been confirmed previously in this species despite being confirmed in other mysticetes [104]. Community monitoring of marine mammals has increased the reporting of *Brucella* in apparently healthy seals harvested for human consumption [100]. 

#### 3.1.2. *Leptospira*

*Leptospira* are a group of zoonotic spirochaete bacteria that have over 200 serotypes described worldwide. Transmission likely occurs through compromised mucous membranes via cuts or wet skin with subsequent dissemination into the bloodstream. In marine mammals, *Leptospira pomona* has been documented in northern fur seals (*Callorhinus ursinus*) in the Bering Sea and in California sea lions (*Zalophus californianus*) as well as in northern elephant seals (*Mirounga angustirostris*) along the coast of California. [105,106,107,108,109]. Since 1970, *L. pomona* has caused both epizootic and enzootic disease in California sea lions [110]. Clinical signs include lethargy, anorexia, polydipsia, reproductive failure, and renal disease. Diagnosis can be via culture of kidney or urine post-mortem, PCR, or direct immunofluorescence on fresh kidneys and urine, or blood samples can be used to assess serum antibody titres. In the Arctic, serology of a small number of bowhead whales has demonstrated a lack of antibodies for *Leptospira interrogans* [111]. 

Currently, the temperate climate favoured by *Leptospira* appears to be limiting the spread of this parasite into the Arctic. In cattle, leptospirosis is most common in late summer, which is correlated with an increase in mean air temperatures [112]. A recent study of livestock in the Russian Arctic has documented 808 cases of this bacteria between 2000 and 2019 in subarctic and Arctic regions [113]. In sea lions, there appears to be a seasonal prevalence pattern, but it is unclear if this is associated with their migratory behaviour or the environmental temperature. However, this is a pathogen whose emergence should be monitored within AMMs [114]. A single bearded seal has been reported with a low titre of 100 to *L. interrogans* in Alaska, and walruses from Alaska have also been found to have *Leptospira* antibodies [115].

#### 3.1.3. *Coxiella burnetii*

*Coxiella burnetii* is a small Gram-negative intracellular bacterium that is the causative agent of Q fever. This zoonotic bacterium can cause flu-like symptoms as well as gastrointestinal symptoms in humans [116]. Despite a wide host range, domestic livestock (sheep, cattle, and goats) are most commonly affected, with weak newborns and abortion or stillbirths being the most common clinical signs. Reports in marine mammals are rare but do exist, as shown by positive serology in sea otters (*Enhydra lutris*), harbour seals, and northern fur seals and the detection of *Coxiella sp* in placenta by PCR in harbour seals, northern fur seals, harbour porpoise, and Steller sea lions [117,118,119,120,121]. Cold Arctic air temperatures have likely limited the spread of Q fever, but it has been documented in cattle in Greenland and is therefore worth further investigation as temperatures in the Arctic continue to warm [122,123].

#### 3.1.4. *Vibrio parahaemolyticus (Vp)*

*Vp* is a Gram-negative bacterium found in marine environments, which has predominantly been studied because of its association with the consumption of raw seafood and gastroenteritis in humans [124]. This organism only proliferates in water of temperatures >15 °C, so its presence in the Arctic has been considered to be an indicator of climate change [125]. It is also an emerging source of infection in the Baltic Sea associated with warming water temperatures [126,127]. The State of Alaska has been screening oysters for *Vp* since 1995. However, it has only been detected since the summer of 2004 when the first human outbreak of *Vp* gastroenteritis occurred in southeastern Alaska. Detection is carried out via culture in trypticase soy agar with 5% sheep blood at 35 °C for 24 h with identification via Vitek 32. Pulsed field gel electrophoresis can further characterise the isolates. While *Vp* has been detected in Alaskan sea otters, harbour porpoises, and white whales, these were an incidental findings and did not appear to have caused significant pathology in any of the infections reported [125]. Studies of the prevalence of *Vibrio* in free-ranging harbour seals off the California coast from 2007–2011 found 29% of animals to be infected [128]. Currently, there are no reports of *Vp* in the Arctic; the most northerly observations are reported from southern Alaska. However, this bacterium has been highlighted as a potential public health risk due to the likelihood of increased prevalence in association with a warmer climate [129]. 

#### 3.1.5. *Streptococcus*

Beta-haemolytic streptococci are one of the most frequently isolated bacteria across all species of marine mammals [130]. Between 2007 and 2012, 1696 marine mammal carcasses (cetacean, pinniped, and sea otter) were screened for the presence of *Streptococcus phocae* in Arctic Canada and the northeastern Pacific [131]. Lung tissue was most frequently positive culture, with 47 of 186 positive cases cultured from the lung. Infection appeared to be opportunistic rather than the cause of death and might be primarily an indication of an underlying pathology. A single case report exists of a uterine infection (pyometra) in a spotted seal harvested by subsistence hunting in Alaska where *S. phocae* was isolated [132]. Streptococcal infections are unlikely to be linked directly to climate change, but the prevalence of infection should be monitored as an indication of overall health status across all AMMs. 

Recently, *Streptococcus lutentiensis* (of the *Streptococcus bovis/ equinus* complex) and *Streptococcus phocae,* known as “*Strep syndrome*”, have been the cause of significant morbidity and mortality in sea otters in Alaska [133]. Additional research is needed, but initial findings demonstrate it is possible that sea otter susceptibility to “*Strep syndrome*” could be associated with previous *Phocine distemper virus* infection and subsequent possible immunosuppression [134,135]. 

#### 3.1.6. *Erysipelothrix rhusiopathiae*

*Erysipelothrix rhusiopathiae* is a Gram-positive bacterium that can present in two forms in cetaceans; a classic dermatological presentation or acute septicaemia, which is often fatal [136,137]. Diagnosis can be via tissue, direct PCR, culture, or via serology. While the disease has been found worldwide and can exist in soil for weeks, it has not been reported commonly in the Arctic, suggesting that cold temperatures may be a limiting factor. However, recently (August 2012), an unusual mortality event in muskoxen (*Ovibos moschatus wardi*) in the Canadian Arctic occurred with *E. rhusiopathiae* [138], demonstrating that this bacteria now occurs in the Arctic and might make its way into the marine environment in the north. Recent reports have documented low levels of prevalence of *Erysipelothrix rhusiopathiae* around the UK in bottlenose dolphins, harbour porpoises, and common dolphins (n = 7/1127) [139], and there was a large scale mortality event in 2021 involving the deaths of 190 harbour porpoises in the Netherlands [140]. To determine any potential increase in incidence, particularly in relation to climate change, it is necessary to continue screening, as routine bacteriology detects most cases even in the absence of gross lesions.

#### 3.1.7. *Pasteurella*

*Pasteurella multocida* and *Mannheimia hemolytica* from the Gammaproteobacteria family are important pathogens in domestic livestock due to their contribution to Bovine respiratory disease, which can cause high morbidity and mortality in cattle [141,142]. Both pathogens have been isolated in cases of septicaemia and mortality in marine mammals [143]. Most members of this family are opportunistic pathogens residing on the mucosal surfaces of healthy individuals. Colonization in compromised individuals occurs primarily within the respiratory tract [144,145]. Diagnosis is possible using multiple methods including bacterial isolation and identification, PCR, and serological tests [146]. As it is primarily an opportunistic pathogen in debilitated individuals rather than highly infectious and transmissible, it is unlikely to cause mass mortality events in AMMs. However, if warming temperatures allow the extension of domestic livestock into the Arctic, then *Pasteurella* would be an example of a pathogen that could be transmitted to wildlife. 

#### 3.1.8. Mycobacteria

Mycobacteria are acid-fast, Gram-positive bacteria in the genus *Mycobacterium,* which are well known because they cause tuberculosis in humans and in other animals. The bacteria are aerobic, rod-shaped, and red in colour when stained with Ziehl–Neelsen, which enables their detection and diagnosis [147]. In marine mammals, *M. pinnipedii* causes tuberculosis in pinnipeds in other parts of the world such as the New Zealand sea lion (*Phocarctos hookeri*) and can be detected and characterised by culture or PCR [148]. *M. abscessus* has been found to cause pneumonia in cetaceans [149]. Mycobacterial infections in seals are not uncommon in temperate climates [150]. Environmental exposure to non-tuberculosis-forming mycobacteria has been confirmed in free-ranging cetaceans with measurable titres reported, although widespread disease as a result of mycobacteria in marine mammals is rare [151]. *M. avium* of the subspecies *paratuberculosis,* which is the cause of Johne’s disease, has been detected in several herds of caribou (*Rangifer tarandus* spp.) in the Arctic. This demonstrates that the bacteria can survive within the Arctic, although it is infrequently reported in AMMs [145]. 

#### 3.1.9. *Nocardia*

*Nocardia* species are Gram-positive facultative intracellular aerobic bacteria that are found ubiquitously in soil and water [152]. The most common presentations are systemic, with pyogranulomatous lesions in multiple organs, most commonly in the lungs. Detection is via culture and via haematoxylin and eosin and acid-fast staining of bacteria. With a 100% mortality rate in marine mammals, this pathogen is of concern [152,153,154,155,156]. However, to date, it has not been observed in the Arctic [154,157,158,159,160]. Ante-mortem diagnosis via sero-diagnostic testing remains challenging [161]. 

### 3.2. Fungi 

More than 320 fungal organisms have been isolated in the Arctic, creating significant potential for fungal disease. However due to their temperate climate preference and the shortage of potential vectors, there are currently no reports of fungal disease as primary pathogens in AMMs [154,162]. Most fungal species that are known to affect marine mammals occur in human care situations, where *Candida albicans*, *Cryptococcus, Fusarium, Blastomyces, Zygomycetes,* and *Coccidioides* have been documented [145,163]. For fungal organisms inhabiting Polar Regions, the main vector of transmission is air [164]. *Aspergillus* is the primary fungal agent of concern because it can cause respiratory infections in cetaceans, and it is an indicator of systemic immunosuppression [154]. It has been diagnosed in both captive and free-ranging cetaceans as far north as Scotland [165,166]. *A. fumigatus* produces a mycotoxin that causes immunosuppression, which in turn increases host susceptibility to other pathogens. Marine strains of *A. fumigatus* have been found in free- ranging cetaceans along with morbilliviruses [165,167,168]. Clinical presentation is frequently abnormal neurological signs and respiratory infection. Accurate serological tests are still being developed. Diagnosis can be via culture but caution should be taken because this organism is present in the environment [163]. No reports currently exist for *A. fumigatus* in AMMs. As fungal diseases in marine mammals can present as opportunistic infections or as indicators of underlying health concerns, an increase in the prevalence of fungal disease could indicate declining health within a population. 

### 3.3. Parasites

Most AMM parasites live within their hosts, although some few ectoparasites do manage to survive on marine mammals. The most common endoparasites in AMMs are helminth parasites, which are commensals to their host and usually do not cause health issues. However, large burdens of parasites can have negative effects either directly or indirectly [169]. Previously benign parasites can become pathogenic in a susceptible host, affecting health and immune status. Studies in the Arctic to date suggest that the influence of climate change on Arctic host–parasite systems will likely favour the parasites rather than the hosts [170,171]. Here, the parasites of primary concern to AMMs and those of potential emerging disease importance are reviewed. 

#### 3.3.1. *Trichinella*

*Trichinella* is a genus of roundworms that can cause zoonotic disease, particularly in the Arctic where transmission to humans can occur from the consumption of raw meat, resulting in serious gastrointestinal symptoms [172,173,174,175,176]. *Trichinella* has been diagnosed in several Arctic species dating back to the 1940s, including walruses in Greenland [177,178,179] and killer whales in Pond Inlet, Canada [180]. Historically, microscopic examination of polar bear diaphragm and masseter muscles has found infection rates of *Trichinella* as high as 58% (n = 364) [181]. More recently, low levels have been reported in polar bears assayed for antibodies against *Trichinella* spp. [182,183]. Interestingly, surveys of bearded seals (n = 84) and ringed seals (n = 252) have shown only negative results [181]. Broader screening should be conducted to identifying which AMMs carry high levels of Trichinella from a subsistence-hunting zoonotic perspective [180,184]. 

#### 3.3.2. Trematodes

Trematodes are parasitic flatworms known as flukes within the subclass Digenea and can cause pathology in marine mammals, primarily affecting the liver and lungs of the host species. The hepatic fluke *Orthosplanchnus arcticus* has been described in ringed seals in Greenland [185] and in a bearded seals in the Chukchi Sea [186]. In other species such as sea lions or common dolphins in California, trematode eggs from *Zalophotrema hepaticum* and *Campula rochebruni* have been found in the brain at necropsy with associated meningoencephalitis [187,188]. The lifecycle is not fully understood, but molluscs or fish are likely intermediate hosts. Diagnosis is predominantly post-mortem as an incidental finding. Recently, faecal analysis has also confirmed the presence of eggs and larvae of trematodes in polar bears [183,189]. 

#### 3.3.3. Cestodes

Tapeworms are extremely common in the intestinal tracts of AMMs, including those of bearded, harp, hooded, ringed, and harbour seals as well as walruses [186,190,191]; some few have larvae that migrate through the abdominal cavity (*Phyllobothrium, Monorygma, Polypocephalus*) [192]. Although they are common in seal intestines, they rarely cause pathology unless they occur at high enough burdens to block the intestinal lumen [193]. Clinical signs of heavy infestations are gastro-intestinally related and can include diarrhoea, anorexia, emaciation, and anaemia. 

#### 3.3.4. Acanthocephalans

Thorny-headed worms within the genus *Bolbosoma* have been found in cetacean intestines [194], and members of the genus *Corynosoma* have been found in northern fur seals and harbour seals in Alaska [195] as well as in other pinnipeds. As with other helminths parasites, a small load can occur in a commensal relationship, whereas a heavy load can be pathogenic. Heavy burdens of these species cause severe ulceration of the intestinal wall [196]. Cases have been reported in stranded cetaceans with obstructive loads of acanthocephalans; this seems to be secondary to malnutrition or poor overall health [194,197]. No acanthocephalans have been reported in polar bears. However, the recent increase in observed helminths in general in polar bears warrants continued monitoring [183,198,199]. 

#### 3.3.5. Nematodes

Roundworms are the most diverse and numerous of the helminth parasites found in marine mammals with a wide range of adaptations resulting in more deleterious effects. Numerous roundworms have been documented in AMMs in the lungs of ringed seals [200], the stomachs of bearded seals [201], and in multiple organs in ribbon and spotted seals [202]. Common nematodes in AMMs include *Ascaroids* such as *Anisakis*, *Spirurids* such as *Crassicauda*, *Filaroids* such as *Acanthocheilonema*, *hookworms* such as *Uncinaria,* and *Metastrongyloids* (lungworms) such as *Otostrongylus circumlitus* [203,204,205,206,207]. Zoonotic *Anisakis* is well documented in both people and AMMs [208]. Lungworm has been reported in all AMM seals [200,202,209,210]. A report also exists of *Metastrongyloidea* found upon necropsy in narwhal, which were captured for public display in 1970 in northwest Canada [211]. With nematodes already prevalent within AMMs, there is the potential for numbers to increase if host susceptibility shifts because of climate change. Therefore, documentation of the presence (or absence) and quantity at necropsy is important.

#### 3.3.6. Parasitic Arthropods

Few parasitic arthropods have been documented in AMMs due to the fact that most are intolerant of both cold environments and marine environments [212]. However, some parasitic arthropods do specialise on marine mammals, and a high burden of parasites can be indicative of underlying health concerns and lower immunity, which leaves the individual susceptible to parasitic infection. Thus, these organisms should be assessed in AMMs where possible. The seal louse (*Echinophthirius horridus*), for example, is one of the few insects that has successfully adapted to the marine environment [213]. The seal louse is itself not dangerous, but it is an intermediate host of seal heartworm *Dipetalonema spirocauda* and *Acanthocheilonema,* which are more likely to have deleterious effects on seal health [214]. Lice have been reported in harbour, grey, harp, and ringed seals and walruses; transmission likely occurs through physical contact among animals [212,215,216]. A nasal mite, *Halarachne halichoeri,* has been documented in spotted seals [217] as well as in walrus [218]. While Arctic seals have been exposed minimally to date, warming temperatures could give rise to increased vector distribution, resulting in increased disease prevalence [219]. 

Whale lice, or cyamids (amphipods that are ectoparasites living on the skin of many whale species), are harmless to the host animal at normal levels, but large numbers can indicate underlying health concerns and low immunity [220]. From 1973 to 2015, 673 bowhead whales were examined for cyamids (*Cyamus ceti*) in Alaska, and they were found to be present at low abundances on 20% of the whales with fewer than 10 cyamids present on 95% of the whales [221]. Cyamid presence was found to be higher in the spring and autumn and in older or physically impaired individuals. In general, the number of cyamids counted has decreased over the last 35 years in Alaska. Unlike right whales (*Eubalaena* spp.) that have callosities that provide a mooring place for cyamids, the absence of callosities on bowhead whales means that cyamids are most often present only when damaged skin provides a place to attach [221]. Whale lice are also common on the other endemic Arctic whales; narwhal and white whale share the same two cyamids, *C. monodontis* and *C. nodosus* [220,222].

Another factor to consider with parasitic arthropods is their ability to transmit disease. For example, *Bartonella henselae* is an intravascular bacterium that is transmitted by arthropods. This organism has been diagnosed as contributing to the cause of death in a captive white whale, as well as being present in two of three tested hunter-harvested white whales [223]. 

Mange in black bears and other mammals is caused by the mite *Sarcoptes scabiei* [224]. It has not been reported in polar bears, most likely due to normal cold temperatures in this species range [225]. However, a recent study has confirmed *Francisella tularensis*—a bacterial infection—in polar bears in Alaska, which is transmitted through the bite of an arthropod vector [226]; the 13% positive seroprevalence in polar bears (n = 83) in this study demonstrates that there is a level of exposure to an unknown arthropod that was previously unrecognised. Expanding host ranges could result in the further expansion of parasites and potential spill-over into novel hosts. 

### 3.4. Protozoan Parasites 

Numerous protozoans infect marine mammals; these can range from normal biota, to host organisms, to pathogens. The primary concern regarding protozoal infections in marine mammals is mortality due to encephalitis, but in theory there is also a potential risk of reproductive failure. This review will focus on those protozoans deemed most important to AMMs. 

#### 3.4.1. *Toxoplasma gondii*

It is currently unknown how AMMs become infected with *Toxoplasa gondii* in the absence of direct contact with oocysts from infected felids (the definitive host). There is no known definitive host in the marine environment [227], though, theoretically, any marine mammal could be an intermediate host. Several studies have demonstrated seropositivity in Arctic species (narwhal, polar bear, ringed seals, and bearded seals) and PCR positive tissues [228,229,230,231]. The most likely route of infection is through the consumption of viable cysts in prey tissue, with differences in prey selection accounting for differences in prevalence between species and between the sexes within species [228,232]. The seroprevalence of 828 Arctic seals in Canada screened for *T. gondii* antibodies was 10.4% between 1999 and 2006 [233]. Terrestrial species are more likely to be infected; a recent serosurvey in Svalbard explored the prevalence in multiple species, finding a 43% positivity in Arctic foxes (*Vulpes lagopus*) followed by 7% in barnacle geese (*Branta leucopsis*) and 6% in walruses [234]. This study in Svalbard also demonstrated the potential for migratory birds to be a source of infection for *T. gondii* in AMMs and other species. Another recent publication hypothesised an alternative route of transmission through hydrological modelling [235]. Snowmelt runoff could be a new source of *T. gondii* infection for marine mammals and subsequently increase the risk of human infection via the consumption of uncooked meat.

The seroprevalence of *T. gondii* infection in polar bears in the western Hudson Bay has increased from 43.8% in the 1980s to 69.6% in 2015–2017 [236]. In this same study in Hudson Bay, it was also noted that there was a higher *T. gondii* seroprevalence following wetter summers. Polar bear blood samples from the southern Beaufort Sea (N = 139) tested for exposure to *T. gondii* showed that the seroprevalence increased with time through the data series [89]. The probability of polar bears being positive for *T. gondii* was seven times higher in individuals that spent more time on land compared to those that resided most of their time on sea ice. 

Warmer water temperatures might play a role in the prolonged survival of oocysts of *T. gondii* in the future, increasing the risk of transport into the Arctic via north-flowing currents such as the North Atlantic Current or though marine invertebrate filter feeders [228]. White whales (n = 27) from Svalbard, Norway, were tested for *T. gondii* but were found to be negative [237], although antibodies have been detected in white whales in the Sea of Okhotsk [238]. Whilst *T. gondii* is currently a low mortality risk in the Arctic, an increased incidence could occur if the overall health of populations deteriorates. A recent study in harbour porpoise in Greenland demonstrated false-positive *T. gondii* results via a direct agglutination test, which was confirmed to be negative via ELISA and PCR. This indicates the need for caution when testing in AMMs, which are often rich in lipids, as this can lead to false-positive results from non-specific adherence to tachyzoites in the direct agglutination test [239,240]. From a human health perspective, there have been outbreaks documented in pregnant women in northern Quebec as well as high levels in wildlife harvested by Inuit communities in this region [241,242,243]. 

#### 3.4.2. *Neospora caninum*

The presence of *N. caninum* antibodies was first reported in marine mammals in 2003 in sea otters, walruses, Californian sea lions, harbour seals, ringed seals, bearded seals, and bottlenose dolphins (*Tursiops truncatus*) [232]. In all of the tested species, the levels indicated exposure rather than active clinical infection. Dogs are the definitive hosts of *N. caninum*, making areas of the Arctic that are more populated at greater risk for transmission [244]. Clinically, *Neospora* is known to cause abortion in cattle and neurological disease in dogs [245,246]. No clinical reports of death due to *Neospora* have been confirmed in studies of this parasite in polar bears, but confirmation would require histopathology of the foetus to confirm the underlying cause of abortion. Such samples would be almost impossible to obtain [236]. Seropositivity for *N. caninum* has been confirmed in polar bears in human care and in the wild [247]. 

#### 3.4.3. *Sarcocystis*

*Sarcocystis neurona* is an important cause of protozoal encephalitis in marine mammals. A wide range of marine mammal species have been confirmed to be affected along the west coast of the USA in addition to some marine mammals in human care [248,249]. Opossums (Didelphis virginiana) are the only known definitive host for S. neurona in the USA, although alternative hosts may exist in the Arctic [250,251,252]. Positive diagnosis is achieved via PCR assay with no clinical signs apparent in the majority of intermediate hosts. S. neurona can cause encephalomyelitis if the parasite migrates from the encysted muscle to the brain [253,254].

In polar bears, *Sarcocystis canis* is of primary concern [255]. *Fatal sarcocystosis* has been reported in captive polar bears in Anchorage [256], but currently it has not been documented in the wild. Temperature and a lack of intermediate hosts has likely prevented its spread in the Arctic. The life cycle of this parasite in bears is not fully understood [257]. Increased interaction between intermediate hosts such as domestic species and polar bears could increase the prevalence of S. canis in polar bears [257].

#### 3.4.4. *Giardia*

*Giardia* is a widespread pathogen that is easily transmitted in water. *Giardia* infections in AMMs are well documented, with reports being obtained from both Alaska and the Canadian Arctic [258,259]. Of 31 ringed seals tested for *Giardia,* 64.5% were positive, and in 39 bowhead whales, 33% were positive [258]. Despite its widespread detection, clinical disease has not been associated with this pathogen. The zoonotic significance of *Giardia* warrants further monitoring, particularly in populated areas [260]. Recently, in beavers (*Castor canadensis*), climate change has facilitated a northern range expansion, which in turn has also shifted the range of *Giardia lamblia,* which is carried by beavers and has also been found in a wider range, increasing the risk of exposure to this parasite in previously unexposed Arctic species [129].

#### 3.4.5. *Cryptosporidium*

*Cryptosporidium* is another ubiquitous protozoan parasite that is detected widely in marine mammals, but it does not appear to cause clinical disease. It has been isolated from the gastrointestinal tracts of ringed seals in Canada [261,262]. A potential pathway for infection is through environmental pollution with human and domestic animal faecal material [263].

#### 3.4.6. *Eimeria*

*Eimeria* causes symptoms of bloody diarrhoea and emaciation in domestic livestock, but infections in marine mammals appear to be primarily incidental and of minimal clinical significance. The exception is in phocids, with harbour seals susceptible as definitive hosts to *E. phocae;* transmission most likely occurs at haul-out sites [264,265]. There is potential for an outbreak in the Arctic given that this protozoa can withstand cold conditions, and reduced habitats could induce higher densities of animals than the norm for species such as ringed and bearded seals and even terrestrial concentrations of some of the ice seals (see [266,267]).

### 3.5. Viruses

#### 3.5.1. *Paramyxoviruses* (including Morbillivirus)

The emerging infectious disease risk of viruses is arguably of greatest concern to AMMs. Morbilliviruses are of primary concern, with phocine distemper virus (PDV) in pinnipeds and cetacean morbillivirus (CeMV) in cetaceans [268,269]. Both have been encountered in AMM species [270]. The monitoring of serum antibody titre levels is of primary importance to assess exposure levels, immunity, and significance as a possible cause of mortality. 

Epizootics resulting in extensive mortality in pinniped species have been documented repeatedly and appear to play a role in regulation of population size in some species of marine mammals, with outbreaks occurring when populations approach carrying capacity [271,272]. Relatively, recent harbour seal (and some grey seal) epidemics in the North Sea in 1988 and 2004 were confirmed to be PDV [273]. The 16-year interval between outbreaks gave the North Sea population sufficient time to recover and reach carrying capacity again. In these outbreaks of PDV, the close proximity of grey and harbour seals was determined to be a primary factor in the disease transmission [273]. As the populations reach carrying capacity and are physically competing for haul-out space, the disease transmission rate is likely to increase [266,274]. 

The mass mortality of polar phocids due to viruses has been suspected as far back as the 1950s [275]. Sea ice declines could lead to increased crowding in haul-out areas, increasing the risk of both intra-specific and potentially even inter-specific transmission [266]. The population levels of Arctic pinnipeds in many regions are high enough to sustain PDV within populations [276]. Although it is unknown exactly what paradigm shifts could initiate the next PDV outbreak, warm temperatures and high population densities are hypothesised to be key factors [277]. The more solitary behaviour of spotted seals, their avoidance of crowded haul-out sites, and close contact with conspecifics may reduce their disease susceptibility [278]. This could also explain why antibodies for PDV or CDV were absent in spotted seals screened between 1998 and 2008 [279]. Phocids demonstrate variable morbidity and mortality to PDV, with North Atlantic harbour seals seemingly being most susceptible to fatal PDV, with >53,000 dead in the 1988 and 2002 outbreaks [280]. However, susceptibility to infection is highest in harp seals (83% of 157) followed by ringed seals (41% of 259), with grey and hooded seals seemingly being less susceptible [270,281,282]. Although there is serological evidence of exposure in walruses in the eastern Canadian Arctic, there is currently no indication that they are susceptible to PDV [281,283].

From 1980 to 1994, a longitudinal study sampling for morbillivirus in grey seals and harbour seals on the east coast of North America found the prevalence of morbillivirus-neutralizing antibodies to be significantly higher in grey seals (73%) compared to harbour seals (37%) with phocine distemper virus as the predominant titre [284]. A 15-year study from 2001–2016 sampled 2530 live ice-associated seals (bearded, ribbon, ringed, and spotted), obtaining paired blood and nasal swabs where possible; for dead animals (n = 165), samples were obtained from tissues and blood [5]. This longitudinal dataset is an excellent example of establishing both baseline data and comparative trends over time. By performing serology, PCR, and sequencing for widespread viral exposure, it was determined that infection with PDV was present. A particularly significant finding in this study was the presence of PDV in the Pacific Northwest, with infection rates increasing as sea ice extent declined. This study suggests a link between climate change and the introduction of PDV in ice seals. 

Clinical signs of morbillivirus infection in pinnipeds include a multitude of symptoms, including lethargy, head tremors, convulsions, and seizures, with impaired swimming and diving behaviour being the most obvious and advanced neurological signs. Clinically, phocids present with serous or mucopurulent ocular and nasal discharge coughing, mucosal cyanosis, pyrexia, and dyspnoea. Pregnant females may abort. In post-mortem examinations of pinnipeds with a suspected viral cause of death, the following organs should be sampled to facilitate screening for morbillivirus, herpes virus, influenza, or parainfluenza: brain, lungs, liver, kidneys, spleen, and lymph nodes [281]. 

Morbillivirus is also of significant concern in cetaceans, with mass mortalities of whales being recorded due to this viral infection [285]. Cetacean morbillivirus (CeMV) has three well-characterised strains: PMV—porpoise morbillivirus; DMV—dolphin morbillivirus; and PWMV—pilot whale morbillivirus [286]. A naïve endemic Arctic species without previous morbillivirus exposure could be susceptible to infection and a high epidemic risk that could result in significant mortality. Transmission is suspected to be through the inhalation of aerosolised virus, although vertical transmission from mothers to their offspring has also been documented [287]. Clinical signs can be associated with acute mortality or the chronic course of secondary infections and chronic encephalitis. Diagnosis is usually post-mortem via histology, virus isolation (the gold-standard), immunohistochemistry, RT-PCR, or serology [288]. Characteristic post-mortem findings include encephalitis, bronchointerstitial pneumonia, and lymphoid depletion. Blood samples from 339 narwhal samples taken from 1984–2003 confirmed antibody presence to morbillivirus [100]. The bowhead whale should be considered at risk given that morbillivirus has been observed in other mysticetes, though no confirmed reports of exposure or disease exist in bowhead whales at this time [289,290]. 

#### 3.5.2. Influenza A Virus

Influenza A virus is a highly contagious virus of the *Orthomyxoviridae* family, which causes acute respiratory disease with high morbidity but low mortality in most species [291]. Serological monitoring has confirmed the presence of influenza A in the Canadian Arctic, with white whales and ringed seals being serologically positive; antibodies have also been confirmed in narwhal and bowhead whales [292,293]. In addition, influenza A antibodies have also been detected in white whales in Svalbard, Norway, and in Russia, though there are no reports of clinical disease [237].

The detection of influenza A antibodies is most often achieved through competitive ELISA (cELISA) using monoclonal antibodies against the influenza A nucleoprotein. In the Nielsen et al. (2001) study including samples from 1984–1998, the virus could not be isolated, and they postulated that infection was likely sporadic and self-limiting in the ringed seals and beluga. However, there have been mortality events associated with influenza A, with 425 harbour seal carcasses on the west coast of Sweden infected with influenza A subtype H10N7 [294]. Similarly, in the US in 2011, 162 New England harbour seals died due to the presence of H3N8 influenza A virus [295]. 

Highly pathogenic avian influenza (HPAI) virus H5N8 has been documented in harbour seals of the German North Sea coast in 2021 in conjunction with the high levels of avian influenza in wild birds in 2020/2021, with the likely route of transmission being through the oral uptake of contaminated bird faeces by seals [291,296]. Infected seals were primarily pups less than six months old clinically presenting with signs of pneumonia. Increasing reports of influenza A outbreaks in marine mammals are of particular concern due to the epizootic and zoonotic potentials of this virus [297,298,299]. In the subarctic, Northwest Atlantic grey seals have been shown to be potential reservoirs; therefore, the increased interaction of seal species in the same habitats could also result in increased viral transmission [300]. While a large-scale outbreak has not been reported in the Arctic as yet, it is possible that with climate-change-driven avian range expansions, cases could increase in the future. The current (2022–2023) HPAI (EA H5N1) has been documented in the Arctic in gulls in Svalbard [301,302]. This virus has also been detected in some terrestrial mammals including red fox (*Vulpes vulpes*), black bears (*Ursus americanus*), and Kodiak brown bears (*Ursus arctos middendorffi*) in Alaska [303]. 

#### 3.5.3. Coronavirus

Isolated cases of coronavirus in live animals and in animals sampled post-mortem have been documented in bottlenose dolphins and in harbour seals [304,305]. In light of the global COVID-19 pandemic, increased screening has been conducted on marine mammals in human care and in free-ranging species. Currently, this virus is not of primary concern for mortality in marine mammals; however, it does present a potential risk to marine mammals should new more pathogenetic strains occur [306].

#### 3.5.4. Calicivirus 

The most commonly found *Caliciviridae* viruses in marine mammals are vesiviruses, which are strains of vesicular exanthema of swine virus (VESV) and include the San Miguel Sea Lion viruses (SMSV). Noroviruses also occur, which are similar to those found in oysters (which can affect humans). Clinical signs of the vesivirus in pinnipeds and cetaceans include epidermal vesicles [307], but lesions appear to heal without intervention. Serum samples from 21/36 harvested bowhead whales in Alaska were found to be positive by virus neutralization for antibodies to VESV and SMSV [308]. Low levels have been documented with seropositive bowhead whales [290]. Walrus faeces collected from sea ice in the Chukchi Sea were also found to test positive for three different calicivirus isolates, and antibodies were confirmed positive to varying calicivirus strains in 7/155 walrus blood samples [309,310]. Similarly, at St Lawrence Island and at Round Island, Alaska, 18% of walrus tested (7/40) showed serological responses to one or more calicivirus [105]. With low morbidity and mortality, calicivirus is deemed to be of little concern to AMMs.

#### 3.5.5. Adenovirus

Adenovirus infection has been documented in both pinnipeds and cetaceans. The *Mastadenovirus* genus has a wide host range in marine mammals, with several documented cases occurring in rehabilitated California sea lions with viral hepatitis being the most common finding [311]. Despite high morbidity, mortality is low. Gastrointestinal samples from bowhead whales have been found to be positive [111]. However, in seropositive bowhead whales, no histopathological lesions where observed [290]. Given that mortality risks due to adenovirus are low, it is currently of little concern. 

#### 3.5.6. Herpesvirus

Alpha and gamma herpesviruses have been detected worldwide in phocids and cetaceans, including species in the Arctic [312,313]. With their wide global distribution and circulation in many populations, latent infections are common in their hosts with reactivation during immunosuppression. Recent research has confirmed the role of herpesvirus in California sea lion cervical cancer [314]. Herpesvirus lesions in marine mammals have been documented to range from genital lesions to fatal herpesvirus encephalitis [315,316]. Phocid seals have been confirmed positive in the northern hemisphere and in the Arctic [317,318]. Mortality appears to be rare.

#### 3.5.7. Papillomavirus

Papillomavirus can present in marine mammals as cutaneous, genital, or lingual warts. However, these viruses are generally regarded as both host- and site-restricted. Reports exist for papillomavirus in white whales, particularly those in the contaminated St Lawrence Estuary, narwhal, and killer whales [319,320,321,322,323]. The presence of papillomavirus rarely impacts health or causes widespread morbidity or mortality. 

#### 3.5.8. Poxvirus

Seal pox presents as single or coalescing nodules that are usually up to 2.5 cm in diameter with characteristic histopathology and confirmation of parapoxvirus particles upon electron microscopy [324]. While the disease itself is not fatal, it does reflect general immune status; widespread poxvirus lesions are likely associated with additional pathology [325]. For example, the immunosuppressive effects of morbillivirus can lead to poxvirus infections [326]. 

#### 3.5.9. Rabies Viruse

Rabies is caused by the *Rhabdoviridae* virus and is spread through bites from an infected animal, with the Arctic fox being the most important viral reservoir in the Arctic [327]. Despite large numbers of marine mammals being tested for rabies, few positive cases have been reported; single positive cases exist for a polar bear and a ringed seal in Svalbard, Norway [328,329,330]. Brains are examined by standard or direct fluorescent antibody test (FAT) for confirmation, and RT-PCR can be used to characterise strains [328,329,330]. An increase in haul-out time or time on land due to climate change could result in an increased exposure of Arctic seals to rabies. Therefore, the detection of any unusual behaviours such as neurological disorders or abnormal locations of species should consider rabies as a possible cause. 

### 3.6. Other Climate-Change-Related Health Stressors

Climate change is likely going to increase various other sources of stress, morbidity, and mortality in AMMs, adding to cumulative risks to their health. Sea ice reductions are increasing accessibility to active shipping lanes and commercial fishing, which could in turn cause anthropogenic trauma from vessel collisions, entanglements, or bycatch and gas embolism syndrome as well as competition for fishery resources being harvested [64,68,331,332]. Studies monitoring the effects of increased shipping activity on AMM health have observed increased cortisol levels in narwhals, suggesting stress [30]. Improved diagnostic testing of cortisol in baleen could also be used to assess cumulative stressors in bowhead whales [333]. Increased fishing and shipping in the Arctic will likely continue with the opening of the Northwest Passage and Northern Sea Route [334,335]. Increased High Arctic shipping could be a particular risk to the slow-swimming bowhead whales that seasonally surface-feed [336,337,338,339].

#### 3.6.1. Interspecific and Intraspecific Trauma 

Increasing species overlaps and reduced foraging habitats for AMMs might result in intraspecific and interspecific competition for resources. Climate change is already resulting in distributional shifts and altered species interactions, changing disease exposure and mortality risk [340]. For example, killer whales are spending longer time during the ice-free season in the Arctic, where they prey on ice-dependent cetaceans and other marine mammals, causing severe lesions and direct mortality [337,341,342,343,344,345,346]. Killer whales have been reported to disrupt narwhal habitat use, which could influence the behaviour and foraging efficiency of narwhal and also reduce population size through increased predation [347,348,349,350]. Similar habitat displacement by bowhead whales has been documented while attempting to minimise the risk of killer whale predation [343].

#### 3.6.2. Freshwater Lesions

Changes in sea temperature and salinity have been observed in Arctic areas due to warming and concomitant impacts such as increases in glacial melt, freshwater runoff, rainfall, and changing ocean currents [351,352]. Recent research has documented negative effects of freshwater exposure in bottlenose dolphins, so it is possible that this will also have consequences for other marine mammals [353,354].

#### 3.6.3. Ice Entrapments

Ice entrapments are a potential cause of mortality for species such as white whales [355] and narwhals [356] or migratory species that are unfamiliar with Arctic sea ice patterns, such as killer whales [357,358]. Rain-on-snow events, including ice tidal surges and winter precipitation, and severe storms can result in unreliable breathing holes due to rapid ice formation preventing breathing access [352,359] as well unfavourable conditions to construct birth lairs [360]. Delayed migrations due to unseasonably warmer weather could also result in entrapment because of unpredictable ice conditions [361]. Increased harvesting of narwhals has been reported in association with entrapments, with up to 629 harvested in a single event in 2008 [361]. 

#### 3.6.4. Malnutrition 

Malnutrition due to a lack of prey availability or increased energetic costs of finding food through increased foraging efforts or decreased energy contents in prey that are not traditional Arctic species could also have indirect effects on AMM health [9]. For example, polar cod (*Boreogadus saida*), a preferred prey species for ringed seals, is ice-dependent during its larval and juvenile stages. The reduction in sea ice availability is already impacting the abundance of this keystone species in some Arctic areas [362,363,364]. Years with little sea ice have been correlated with low body condition indices in adult female ringed seals and low reproductive success [365,366]. Novel methods of assessing blubber such as proteomics and blubber metabolomics could improve our understanding of the relationship of blubber depth to nutritional status and overall health [367,368,369]. Malnutrition could increase host susceptibility to all of the pathogens reviewed above [19]. 

#### 3.6.5. Alopecia

Extensive hair loss in seals has been observed in the Arctic intermittently, including during the recent Arctic UMEs [41]. In neonates, extensive alopecia could be a genetic anomaly, whereas in older animals, it could result from an abnormal moult or opportunistic bacterial or fungal infection. Moult can be impaired by underlying pathologies in addition to stress or poor nutrition [370]. In addition, moult is also dependent upon access to ice platforms for haul-out, so abnormalities could be associated with climate change reducing ice availability [371,372]. 

#### 3.6.6. Neoplasia

While neoplasia is generally a non-infectious disease, increased trends in this disease can be indicative of overall health status in AMMs. Sporadic reports exist in the literature of neoplasia in AMMs including pulmonary mast cell tumours in walruses [373], brainstem carcinoma in white whales [374], and adenocarcinoma in ringed seals [375]. In over 1800 cetaceans examined from 1973 to 1987 from stranding networks in the USA, the diagnosis of tumours was very low (14/1800), with fibromas being found to be most numerous [323]. Infectious agents and chemical exposure have been documented to increase carcinogenesis in some species [376,377,378]. The current lack of neoplasia in AMMs is interesting and should be monitored closely in the future for signs of increased occurrence [322]. White whales in the Gulf of St Lawrence have demonstrated higher-than-expected rates of neoplasia, most likely due to environmental contamination [379].

## 4. Discussion

Infectious-disease-induced mass mortality events are likely to increase in the Arctic as climate change proceeds, with particular risk from bacterial, protozoal, and viral disease vectors. *Brucella*, morbillivirus, Influenza A viruse, and *T. gondii* are likely to pose the greatest risks to AMMs because they have the potential to cause high levels of mortality. These pathogens are already found in the Arctic and are likely to become more prevalent in the future when air and water temperatures becomes warmer and sea ice habitats are further reduced. The relative naiveté of endemic Arctic species to these disease vectors makes them a particular concern. In the recent ice-seal UME, peaks in mortality were noted in the summer, potentially linked to increases in temperature; however, further research is needed to determine the underlying cause (https://www.fisheries.noaa.gov/ accessed on 5 November 2022). 

Whilst many of the other pathogens highlighted in this review are likely of minimal concern to AMM morbidity and mortality independently, with climate change catalysts reducing body condition and resistance, they could act synergistically to result in deleterious health effects [380,381,382]. From a conservation perspective, understanding the epidemiology of disease in AMMs can aid in the allocation of resources and determine the capacity of monitoring required to effectively manage populations. Screening species for the presence of several pathogens simultaneously is likely the most efficient monitoring method moving forward [238]. Active sampling, such as blood sampling for serology titres, will allow the tracking of pathogen prevalence, particularly in the same populations over time. Table 1 provides some of the biological sampling options and pathogen analyses currently available. 

A targeted assessment to determine climate change linkage with the pathophysiology of diseases in AMMs is required [383]. To achieve this, improved systematic monitoring to obtain baseline species information will be vital [383]. Stock assessments can be broadened to detect changing ranges, prey shifts, and health- or body-condition parameters [384], enabling the detection of changes associated with climate change [8], which can influence disease exposure. Establishing disease screenings for AMMs across regions will allow for an improved documentation of the current situation and will permit tracking trends in the future [8,94,385,386]. Marine mammal health (condition, population sizes, and disease status) is a good proxy for marine ecosystem health; the systematic monitoring of these sentinel species in the context of climate change has been recommended repeatedly by Arctic Council working groups (CAFF, AMAP, and PAME) as well as in the scientific literature [11,19,382,387,388]. Specific cases of climate change impacting marine mammal health have been documented involving North Atlantic right whales, Hawaiian monk seals *(Neomonachus schauinslandi*), and California sea lions [383]. But currently no disease studies in the Arctic, where environmental change has been greatest, are definitively linked to climate change, although sea ice declines have been proposed as causal agents of disease outbreaks with both direct and indirect effects expected for all AMM species [42,389,390]. 

AMMs that spend some time on land will likely experience deleterious health effects most rapidly. We already know that prolonged land usage in polar bears subsequent to losses of sea ice has been associated with increased immune reactivity with greater total white blood cell counts as compared to bears that remain on sea ice over the summer period [359,391,392]. Onshore habitat use has also impacted polar bear faecal microbiota and has also increased their proximity to humans, and both factors could influence their pathogen exposure [393,394]. In addition to the aforementioned pathogens, polar bears are also at risk of canine pathogens, with previous serological testing finding positive titres to canine distemper virus, canine adenovirus, canine morbillivirus, and canine parvovirus in Canada [236,395]. Both Arctic seals that haul-out terrestrially and ice-associated AMMs when faced with a shortage of ice such that they are forced to use terrestrial environments could experience similar situations, with increasing contact with people and dogs and other typically more southerly species [266].

The vast areas with low human population densities in the Arctic make the monitoring and physical health assessment of AMMs challenging. Although it is possible for research programmes to target some pinnipeds and smaller cetaceans via live-capture health-assessment programmes, most health data from AMMs are likely to come from subsistence-hunt monitoring. Successful collaborative health assessments can be managed by local communities, particularly those with traditional harvests of AMMs and concerns regarding zoonosis [396]. Carcass-recovery programmes for stranded or deceased marine mammals in the Arctic, where practical, would allow for the increased monitoring and surveillance of current disease states. Increased diagnostic testing of meat harvested for human consumption could also enable the monitoring of pathogen emergence [397]. Extensive health assessments have been performed on subsistence-hunted bowhead whales in Alaska in collaboration with indigenous communities, resulting in a rich health database [398,399,400]. The monitoring of even small-scale sports hunts, such as the ringed seals shot annually in Svalbard or walrus in Greenland and Canada, can provide an opportunity for health screening across age demographics [401,402]. Documentation of the presence or absence of pathology is imperative to establish baseline health information.

In addition to hands-on examinations, remote visual monitoring could potentially enhance the health monitoring of AMMs in the future. Observations that can inform health status could be achieved remotely, e.g., by drone use for collecting respiratory exudate, data on body condition, and rake marks [403,404,405]. Abnormal respirations can be indicative of lung disease, one of the primary pathologies in compromised marine mammals [145,406]. In pinnipeds, this may manifest as nasal discharge and can be apparent via visual monitoring; in cetaceans, mucous discharge can be harder to assess, though close proximity observations of an increase in mucous or odour can be indicative of lung pathology [407]. Body condition scores can be assessed remotely with drones using photogrammetry [403]. A decreasing body condition score can provide an indication of poor nutrition quality or prey availability or generally compromised individual health status [408,409]. 

Discussing the effects of contaminants on health status is beyond the scope of this review; however, contaminants should be considered in the context of consequences of multiple stressors on individual populations with documented deleterious additive effects in relation to population health status [19,410,411]. Recent publications exploring the immune status of walruses in Svalbard and the effects of contaminants on their health status demonstrate the complex interplay of factors and the need for directed scientific study to answer the question regarding the impact of climate change impact health [412,413]. Similarly, it is well documented that the poor health observed in the St Lawrence white whale population was primarily due to contaminants in this area some decades ago, and that when the environment was improved, white whale health also improved [379]. In pinnipeds, health concerns specific to Baltic ringed seals (and grey seals) include renal pathology, which is proposed to be due to high organochlorine pollution in this specific geographic area [414]. Populations with ongoing contaminant stressors could therefore be more susceptible to the impacts of climate change. 

This review focussed on diseases and risks via pathogen classification, but analysis of the population-specific concerns is especially warranted for successful conservation management. For example, differences within species such as between migratory and non-migratory populations means they are subject to different disease risks, and thus health assessments need to be tailored accordingly while remaining general enough to allow for circumpolar comparisons [238,283,415,416]. A coordinated disease surveillance approach could include community-based monitoring, systematic health data collection, and analysis for specified species and locations, along with longitudinal data on oceanographic conditions such as ice cover, temperature, salinity, and chemistry from regional or global observing systems. These data could be combined with ecological studies of AMMs to enable the development of an environmentally focussed AMM health map [388,417]. Advances in modelling approaches to predict species movements as the sea ice shifts will be key to predicting disease epidemics [418]. Many of the diseases discussed as potential pathogens that could cause AMM morbidity and mortality are zoonotic [419]. A decrease in AMM population numbers or increased disease status could impact subsistence hunting and indigenous lifestyles. With the reliance on marine mammals for local human communities, it is important from a One Health perspective that an interdisciplinary approach is taken to monitor marine mammal health. Monitoring AMM health is vital to good conservation and management. 

## 5. Conclusions

Sea ice declines and concomitant changes in ocean temperatures could be a critical catalyst in the emergence of infectious diseases in AMMs. Recent publications on climate change and marine mammal health demonstrate the gravity of the impact of changes in the environment on marine mammals [50,383,420,421]. Unfortunately, the lack of systematic monitoring for disease has resulted in knowledge gaps regarding the implications for the health status of AMMs. Pathogens of concern are likely going to vary according to species; however, viral pathogens such as morbillivirus appear to represent the greatest disease and mortality risks to AMMs. Bacterial pathogens, such as *Brucella,* are likely going to expand their geographic ranges as migratory species expand northward, increasingly overlapping with AMMs. The gap in knowledge between available baseline data and predicted outcomes in association with climate change needs to be filled by a systematic interdisciplinary approach to facilitate the effective conservation of AMMs. This review highlights methods for monitoring important potential pathogens and identifies which species to prioritise in the establishment of health assessment programmes, creating a map to monitor AMM health. 

## Figures and Tables

**Table 1:** The primary pathogens of concern for each Arctic marine m ammal group with the recommended sampling approach for pathogen detection. Key: 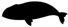 = cetacean, 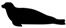 = pinniped, 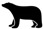 = polar bear. Abbreviations: PCR = polymerase chain reaction, cELISA = competitive enzyme linked immunosorbent assay, RT-PCR = reverse transcription PCR.

Pathogen	Primary Taxa of Concern	Live Animal Surveillance/Monitoring Method	Post-Mortem Organ/Tissue Collection	Sample Storage	Diagnostic Test
*Brucella*	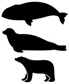	Serum sample	Brain, lung, lymph node, placenta, testis	Frozen −80°	Serology, PCR, prolonged culture on Farrell’s medium, immunohistochemistry
Influenza A virus	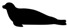	Nasal swab/rectal swab	Blood/tissue: lung, brain, lymph node	Frozen −80°	cELISA, agar gel immunodiffusion, virus isolation, PCR with sequencing
*Leptospira*	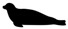	Serum, urine	Blood, kidney, urine	Cold storage	Serology—M AT culture, direct immunofluorescence. PCR
Morbillivirus	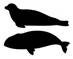	Serum	Blowhole swab, nasal swab, brain lung, lymph node	Frozen −80°	cELISA Virus neutralisation test Real-time PCR Virus isolation and sequencing
Myco-bacterium	* 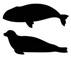 *	Serum	Lymph nodes, sputum, tubercles, swabs.	Frozen −80°	Ziehl–Neelsen staining, prolonged culture, PCR
Rabies viruse	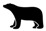	Serum	Whole brain (cerebellum, brain stem, hippocampus)	Fresh mounted tissue, fixed or in paraffin	Antigen fluorescent antibody test RT-PCR
*Toxoplasma gondii*	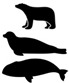	Faeces/EDTA whole blood/urine	Blood serum Fresh/frozen tissue	Ship cold mounted slides or frozen tissue	Modified agglutination test, serology, PCR

## Data Availability

Not applicable.

## Supplementary Materials

The following supporting information can be downloaded at: https://www.mdpi.com/article/10.3390/pathogens12070937/s1, Table S1: Index list of 35 pathogens included in this review.
