# Supplementary material for: A Review of Circumpolar Arctic Marine Mammal Health—A Call to Action in a Time of Rapid Environmental Change
Source: Pathogens. 2023 Jul 14;12(7):937. doi: 10.3390/pathogens12070937 (PMC10385039; doi:10.3390/pathogens12070937)
Supplement: Supplementary file 1 [file pathogens-12-00937-s001.zip › pathogens-2370652-supplementary.pdf]

Table S1—Index list of 35 pathogens included in this review.

1. **Bacteria (n=8)**:
    a. *Brucella*
    b. *Coxiella burnetii*
    c. *Leptospira*
    d. *Vibrio parahaemolyticus*
    e. *Erysipelothrix rhusiopathiae*
    f. *Pasteurella*
    g. *Mycobacteria*
    h. *Nocardia*
2. **Fungal (n=3)**
    a. *Aspergillus*
    b. *Candida albicans*
    c. *Cryptococcus*
3. **Helminth Parasites (n=5)**
    a. *Trichinella*
    b. *Trematodes*
    c. *Cestoda*
    d. *Acanthocephala*
    e. *Nematoda*
4. **Parasitic Arthropods (n=3)**
    a. Seal louse (*Echinophthirius horridus)*
    b. Cyamids (*Cyamus ceti*)
    c. *Sarcoptes scabiei*
5. **Protozoan Parasites (n=6)**
    a. *Toxoplasma gondii*
    b. *Neospora caninum*
    c. *Sarcocystis – Sarcocystis neurona*
    d. *Giardia*
    e. *Cryptosporidium*
    f. *Eimeria*
6. **Viral (n=10)**
    a. Paramyxovirus
    b. Morbillivirus
    c. Influenza A viruse
    d. Coronavirus
    e. Calicivirus
    f. Adenovirus
    g. Herpesvirus
    h. Papillomavirus
    i. Poxvirus
    j. Rabies virus
